# Revolutionizing hyper spectral image denoising: a squeezenet paradigm

**DOI:** 10.1038/s41598-026-36479-1

**Published:** 2026-02-05

**Authors:** Nandhagopal Nachimuthu, Ramya Murugesan, M. Dharmalingam, G. Prakash

**Affiliations:** 1 Professor, Department of Computer Science and Engineering, Nandha College of Technology, Erode, 638052 India; 2 Department of Electronics and Communication Engineering, Karpagam Academy of Higher Education, Coimbatore, 641021 India; 3 Department of ECE, Kongunadu College of Engineering and Technology, Thottiam, Tamilnadu 621215 India; 4 Professor, Department of Biomedical Engineering, Excel Engineering College, Komarapalyam, Tamilnadu 637303 India

**Keywords:** Hyper spectral image denoising, SqueezeNet, Deep learning, Remote sensing, Neural networks, Gaussian noise, Engineering, Mathematics and computing

## Abstract

Hyperspectral images (HSIs) frequently experience various types of noise due to atmospheric interference and sensor instability, which impairs the efficiency of subsequent operations. Consequently, HSI denoising has emerged as a crucial component of HSI preprocessing. Conventional approaches often target a single kind of noise and eliminate it repeatedly, which has disadvantages including inefficiency when handling heterogeneous noise. Lately, models based on deep neural networks have shown encouraging results in the general image denoising domain. This study, which aims to overcome shortcomings in previous techniques, provides a novel denoising methodology by leveraging the effectiveness of the SqueezeNet model. For a thorough assessment, the evaluation framework includes four main indicators: PSNR, SSIM, SAM, and ERGAS. The evaluation is based on real-world hyperspectral images from the [Harvard Hyperspectral Dataset], which cover a variety of scenarios and illumination circumstances. Fire blocks are used by the SqueezeNet-based denoising model to optimize feature extraction with fewer parameters.Benchmarks for comparison include deep learning technique QRNN3D and classical techniques like ITSReg and BM4D.In order to avoid convergence to suboptimal local minima and to speed up and stabilize the learning process, this work presents an incremental training policy. The suggested SqueezeNet-based HSI denoising model performs exceptionally well, attaining competitive results in terms of PSNR of 34.15, SSIM of 0.92, and SAM of 4.56 in addition to impressive ERGAS of 20.47. This study offers an effective denoising solution for hyperspectral images by addressing shortcomings in current techniques, showcasing improvements in efficiency and accuracy.

## Introduction

HSI offer far more detailed data regarding scenes than RGB images since they are composed of large discrete wavebands for every spatial point of real sceneries.HSI consist of numerous bands covering a broad-spectrum range^1^. Unlike conventional color images, HSIs partition the spectrum into a significantly larger number of bands, surpassing the three used in common RGB images. Additionally, the wavelengths in HSI can extend beyond the visible spectrum. HSIs encompass a wealth of both spatial as well as spectral details within a scene, rendering them valuable for numerous purposes^2^. HSI sensors are commonly used in remote sensing applications and are typically deployed on aircraft, satellites or drones, exposing them to challenging operational conditions. New strategies for ultrafast optical sensing and AI-augmented spectral modelling illuminate ways deep feature learning can extract complex carrier–photon interactions and investigate detailed measurements across the spectral region^3–6^. These advances in deep learning applied for imaging plasmonic and hybrid nanostructures highlight the significance of efficient architectures for the real-time reconstruction of signals^7–9^. As a result, noise originating from several sources, including air absorption, temperature, illumination, and sensor failures, can easily contaminate high-sensitivity imaging (HSI) throughout the acquisition process.There are several different types of noise that can be caused by these environmental factors and sensory malfunctions: (i) Gaussian noise (GN), (ii) stripe noise (SN), (iii) dead pixel noise (DN), (iv) impulse noise (IN), and (v) a combination of all these noise types^10^. Furthermore, different kinds and intensities of noise may be present in each band of the HSI^11^.

Due to these influences, the counts of photon in each band are significantly lower than in RGB images, and different types of noise can readily be produced during the acquisition process.This type of degradation has a detrimental effect on all downstream HSI applications’ performance in addition to the HSI’s visual appeal^12^. The presence of noise in the data significantly impacts the performance of image processing and consequent deployments. However, excessive noise frequently corrupts the great majority of HSIs during the generation and transmission process, hence denoising techniques are essential for accurately evaluating and understanding the images^13^.

To address these problems, image denoising typically serves as a noise removal preprocessing step before HSI data evaluation^14^. Reliable results in tasks like target detection, unmixing, and classification depend on this. Nevertheless, a lot of methods used for HSI denoising depend on strategies that initially emerged for RGB or grayscale images, ignoring the abundance of spectral information that exists in every HSI pixel^15^. Furthermore, the data is processed band-by-band using conventional 1-D or 2-D convolution kernels in normal HSI denoising methods. As a result, they ignore the data within the bands, that are essential for the study of spectral signatures, and only consider the spatial information.For example, existing techniques like weighted nuclear norm minimization (WNNM)^16^, block matching and 3-D filtering (BM3D)^17^ are employed to HSI images by analyzing each band as a 2-D image, resulting in significant spectrum aberrations.

The proposed work’s primary contributions are as follows:

- To provide a novel method for denoising hyperspectral images that makes use of the effective SqueezeNet model.
- To utilize the four primary indicators (PSNR, SSIM, SAM, and ERGAS) to conduct a comprehensive evaluation of denoising efficacy.
- To create a standard for hyperspectral image denoising by contrasting the suggested approach with the most advanced methods already in existence.

The remainder of the paper is organized as follows. We examine related HSI denoising techniques and DL strategies in Section II, as these enhance our work. The SqueezeNet method for HSI denoising is introduced in Section III. In Section IV, extensive experimental results on HSI databases are presented. In Section V, conclusions are drawn.

## Literature review

To solve the shortcomings of traditional approaches, deep learning offers a substantial end-to-end learning strategy. Numerous deep learning (DL) approaches have been introduced for HSI denoising. In the initial stages, approaches initially designed for RGB or grayscale images were employed for denoising of HSI, wherein adjustments were made to the input and output filter dimensions or considering them as a single band.

In order to address the problem of maintaining intrinsic similarity in spectral and spatial dimensions, Li et al.^18^ introduce a Spatial-Spectral Transformer (SST) for HSI denoising. To efficiently capture spectral and spatial correlations, the suggested Transformer combines global spectral self-attention and non-local spatial self-attention. Although the spectral self-attention takes long-range dependencies among strongly correlated bands into account, the window-based spatial self-attention investigates spatial similarity across nearby regions. Modern HSI denoising methods are surpassed by the SSTin terms of quantitative measures and visual outcomes, according to experimental data. The efficacy of the method is ascribed to a denoising module that combines spectral attention with window-based spatial self-attention to provide subtle denoising through the weighting of coarse spatial variables with spectral attention.

De Oliveira^19^ presents the Non-local Convolutional Neural Network Denoiser (NL-CNND), a hyperspectral image denoising technique. The technique uses BM4D for a preliminary denoising step and leverages information from four bands adjacent to the target band for enhanced restoration. A CNN is trained on all bands matched with their pre-denoised equivalents in the next step. Especially, the network can adapt to varying noise levels, which improves generalization in various contexts. The approach outperforms the outcomes of BM4D alone. AVIRIS and ICVL images are combined in the training dataset to demonstrate the superior performance of NL-CNND over BM4D and Neural Networks, backed by extensive metrics such as PSNR, SSIM, and SAM.

With an emphasis on hyperspectral images, Gkillas et al.^20^ tackle the problem of calculating sparse representations for multi-dimensional visual data. The study presents a new sparse coding optimization problem with features that make it computationally efficient. In order to overcome the optimization challenge, deep equilibrium and deep unrolling algorithm were created, which result in interpretable deep learning-based architectures. Interestingly, these designs outperform previous sparse coding techniques. This study provides a comprehensive approach for addressing locally dependent signals in multidimensional datasets, establishing a novel link between traditional sparse representation theory and contemporary DL technologies. Training and validation are conducted using the ICVL dataset.

With their innovative Subspace-based Multidimensional Sparse (SMDS) model, Xiong et al.^21^ offer a ground-breaking method for HSI denoising. The SMDS model, which captures important physical meanings, is formulated under tensor notation and emphasizes spectral low-rankness, spatial redundancy, and spectral-spatial correlations. The SMDS model is then optimized and denoising processes are smoothly integrated into an end-to-end network called SMDS-Net. This method teaches precise physical interpretations, in particular, low-rankness and sparsity of HSIs. Comparing SMDS-Net to the most advanced HSI denoising techniques, comprehensive testing on both artificial and real-world datasets has shown that it has strong denoising skill, learning capabilities, and excellent interpretability.

Pang et al.^22^ introduce TRQ3DNet, a novel deep neural network amalgamating CNN as well as Transformer for effective denoising of HSI. The architecture comprises two sections: one featuring 3D quasi-recurrent blocks for spatial and spectral correlation extraction, and the other incorporating Uformer blocks utilizing window-based multi-head self-attention (W-MSA) and locally enhanced feed-forward network (LeFF) for local and global spatial features exploration. A bidirectional integration bridge (BI bridge) is devised to fuse the features from both branches, ensuring optimal preservation of image feature information. Experiments conducted on simulated and actual HSI datasets show that TRQ3DNet performs better. The model’s computational intensity may encounter difficultiesfor real-time operations in resource-constrained scenarios.

The training paradigm was created by Aetesam et al.^23^ with an emphasis on the function of loss functions in neural networks. Additionally, priors based on Bayesian motivation for loss functions limit the solution space to the kinds of noise that are seen throughout the hyperspectral image acquisition procedure. Consequently, denoising performance is improved when loss functions developed in a Bayesian context are used in neural network training. A thorough examination and experimental findings on genuine and artificially tainted hyperspectral datasets indicate that the suggested method may be useful in a variety of noisy, homogeneous, and heterogeneous environments.PSNR and SSIM are computed spatial metrics used for quantitative evaluation.

Outside of hyperspectral image reconstruction, recent works combining AI with plasmonics demonstrate the unique achievement of adaptive optical learning and sub-picosecond charge transfer, emphasizing an increasingly synergistic relationship between photonics and neural computation^24,25^. These preliminary reports emphasize the importance of structures that are compact and high performing such as SqueezeNet, especially in preserving spectral fidelity while being low computationally demanding.

### Research gap

While the current approaches for hyperspectral image (HSI) denoising have made progress, there is still a strong demand for more research and development. These techniques, each with their own advantages, have shown encouraging denoising capability. The hyperspectral imaging field is complicated, nevertheless, and there are still issues with handling different noise levels, improving interpretability, and efficiently collecting intrinsic similarity across spatial and spectral dimensions. Consequently, the goal of the proposed study is to provide a contribution by presenting a method that builds on the advantages of current approaches, thereby bridging gaps and improving the performance of HSI denoising. The proposed methodology aims to give a comprehensive and resilient solution that can be applied to many noise settings, bringing about improvements in terms of accuracy, effectiveness, and interpretability.

## Methodology

### Keywords

A linear model can be used to depict an HSI Y that is subject to various types of noise.

1 $${\mathrm{Y}}={\mathrm{X}}+{\mathrm{N}}$$

wherein X denotes the clean and noise-free image, and N represents the additive noise, such as Gaussian noise. Y, X, N$$\:\in\:{\mathcal{R}}^{B*H*W}$$ are dimensions, with H, W, and B denoting the height, width, and number of bands in the HSI, respectively. The goal of HSI denoising is to recover the noise-free image X from its noisy counterpart.

### Simulation scenario

#### Dataset

**Fig. 1:**
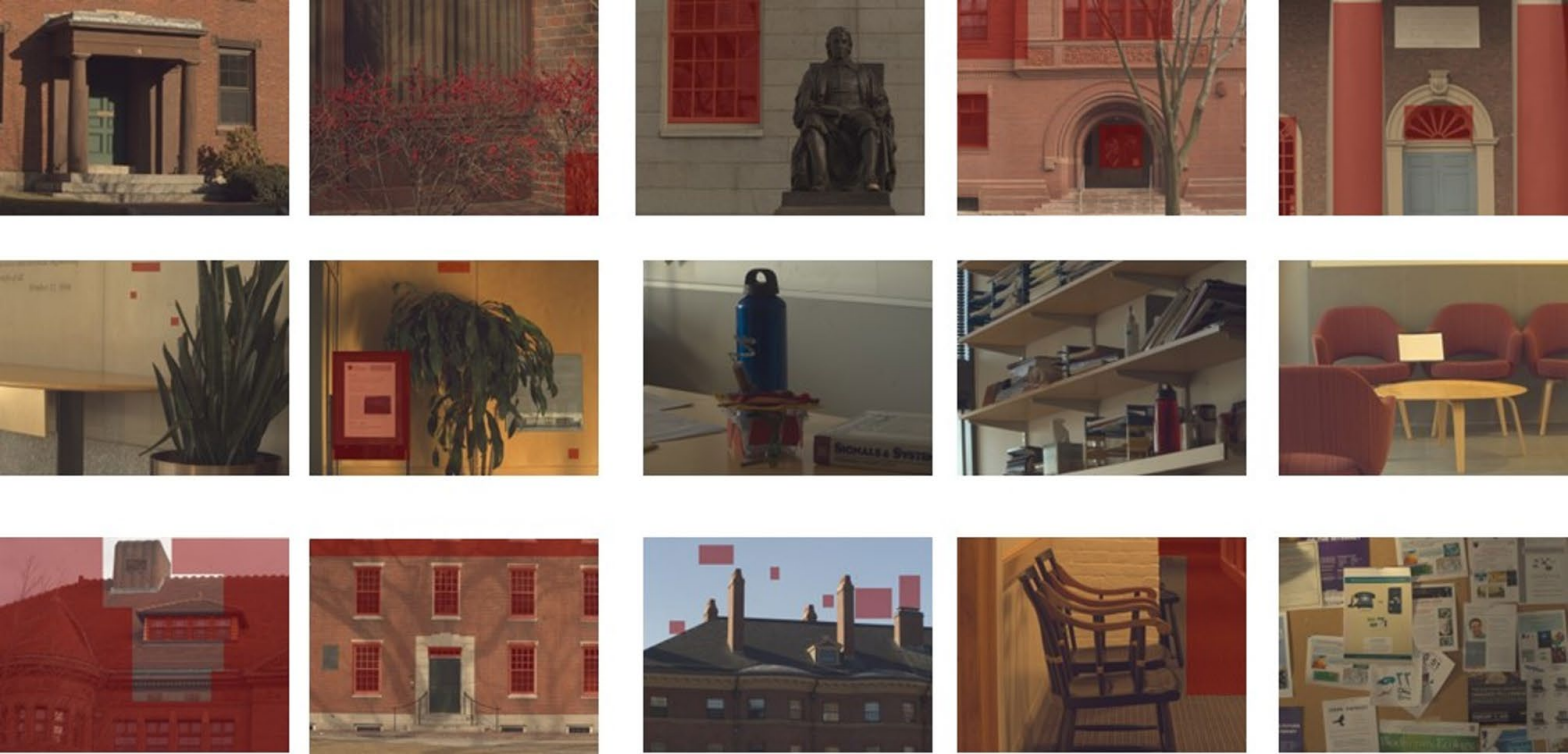
Sample images from the dataset.

This study makes use of real-world hyperspectral images that were downloaded from http://vision.seas.harvard.edu/hyperspec/^17^. This database includes fifty hyperspectral images taken in daylight of both interior and outdoor scenes. In addition, artificial and mixed lighting was used in twenty-seven interior images. Nuance FX, CRI Inc., a commercial hyperspectral camera, was used to capture the images. With the use of an embedded liquid crystal tunable filter, the camera can capture hyperspectral images by gradually adjusting the filter throughout thirty-one small wavelength bands, each having a bandwidth of roughly 10 nm and increments of 10 nm spanning 420 nm to 720 nm. Figure 1 shows samples of developed pseudo-color images from this dataset.

#### Proposed methodology

**Fig. 2:**
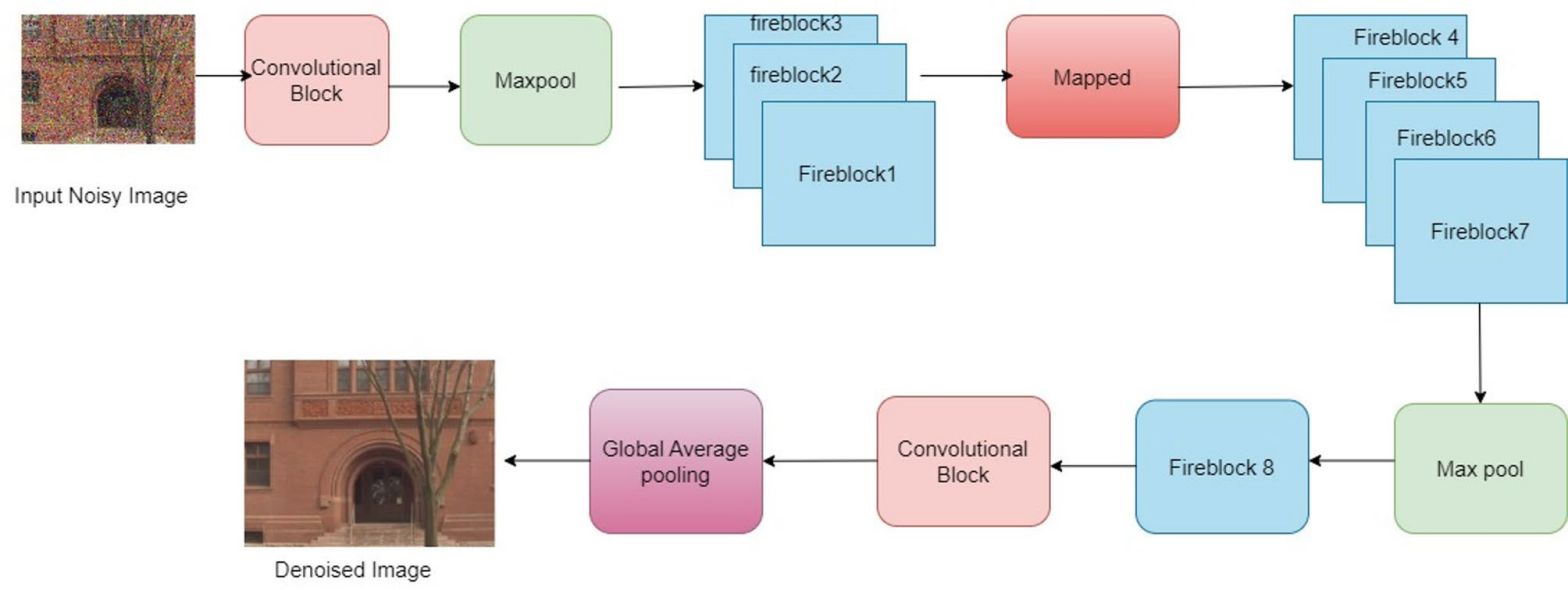
The HSI denoising model based on SqueezeNet.

The input images from the dataset are denoised in order to eliminate the noise from the images. By doing this, the image quality is improved and performance is enhanced. The suggested work uses the SqueezeNet^26^ paradigm to accomplish denoising. With improved extraction capabilities, this model is an advancement over the conventional CNN.Fire blocks are a component of the SqueezeNet architecture that improve the network’s capacity for extraction. Here, the maxpooling process is executed after the input is processed by the first convolution layer, which also extracts the salient features. Subsequently, the fire blocks receive the characteristics, which are then used to discriminate between them in order to detect the noisy pixels. The SqueezeNet model’s ability to retain competitive accuracy while using fewer parameters makes it a superior option over the conventional CNN. As a result, the network requires less time to train and operates more efficiently overall. Figure 2 shows the SqueezeNet-based HSI denoising framework used in this study.

SqueezeNet was chosen after initial tests using MobileNet-V2 and ShuffleNet-V1. While these architectures also provided small structures, their depth-wise separable convolutions resulted in fragmentation of spectral information across bands. Specifically, SqueezeNet’s fire modules are able to keep continuous spectral and spatial coupling, while keeping a model size close to 1 < MB (0.47 MB, to be exact). This combination of compact architecture and representative fidelity made SqueezeNet a particularly good candidate for exploring high-dimensional HSI.

When applied to high-dimensional hyperspectral data, contemporary lightweight CNNs and transformer architectures like MobileNet, ShuffleNet, and SST usually demand more memory bandwidth or processing power than SqueezeNet, despite their high accuracy. SqueezeNet’s fire modules’ 1 × 1 and 3 × 3 convolutional combinations enable effective joint spectral-spatial correlation extraction with sub-megabyte parameter sizes. For real-time airborne or satellite HSI processing, this compactness is essential because it allows for faster training and inference on platforms with limited resources. SqueezeNet’s linear convolutional operations make it more appropriate for large-bandwidth hyperspectral scenes without sacrificing structural fidelity, in contrast to transformer-based techniques that depend on quadratic self-attention complexity.

**Fig. 3:**
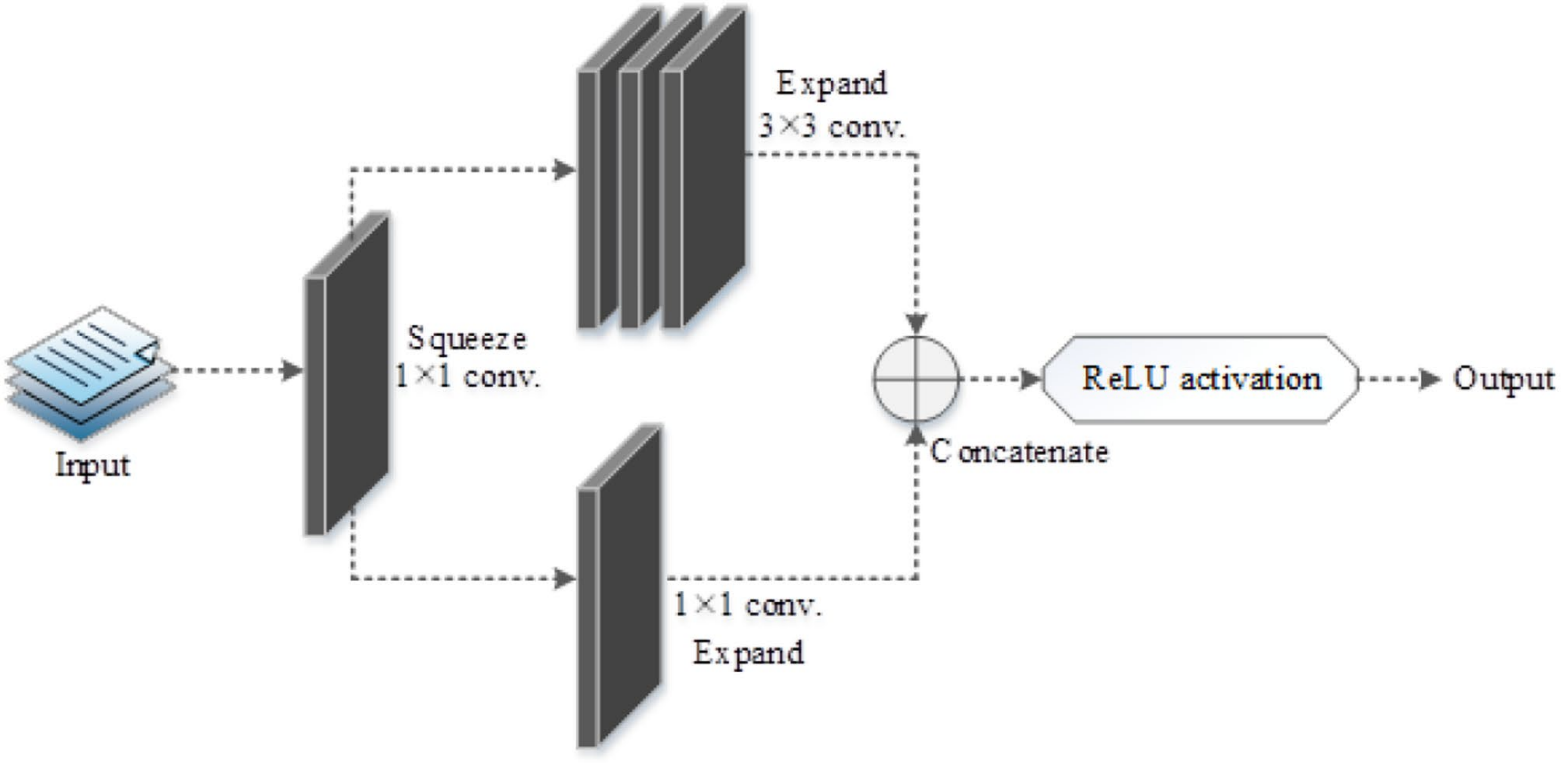
Fire Block structure in SqueezeNet showing squeeze and expand layers. The 1 × 1 convolution layer compresses feature maps (squeeze), while parallel 1 × 1 and 3 × 3 convolutions in the expand layer restore spatial and spectral features before concatenation.

Two convolutional layers with varying numbers of convolutional kernels, eight fire blocks, three max pooling layers, and one global average pooling layer at the end make up the SqueezeNet architecture. SqueezeNet employs three primary ways to minimize parameter counts while preserving improved accuracy. The count of input channels is decreased to 3 × 3 filters in this model, and the 3 × 3 filters are swapped out for 1 × 1 filters. Here, delayed down sampling is used to keep the convolutional layers’ activation maps huge. The goal of this stage is to increase accuracy overall.In the SqueezeNet model, the fire block is made up of squeeze and expand layers having convolutional filters and is regarded as a fundamental unit. Figure 3 depicts the fire block model.

Activation of the rectified linear unit (ReLU) is done throughout the model^27^. The squeeze layer employs the 1 × 1 filter to the input as it approaches the initial fire block, extracting the important features. Subsequently, the 1 × 1 as well as 3 × 3 filters are applied by the expand layer, producing a deeper and more expansive output. While the feature size remains unchanged, the expand function increases the depth, the squeeze operation accomplishes compression. The expand layer’s outputs are then transmitted onto the subsequent blocks after being concatenated.To obtain the final denoised image, the squeeze and expand processes are done over and over for each fire block. The outcome is then transmitted to the global average pooling layer. Equation (2) provides the mathematics for the squeeze action performed in the fire block.

2 $$\:{S}_{qz}\left(y\right)=\sum\:_{\mu\:=1}^{FeM}\sum\:_{i=1}^{Ch}{{wt}_{i}}^{s}{{xn}_{i}}^{\mu\:}$$

where $\:{{wt}_{i}}^{s}$ is the weight connected to the $\:{i}^{th}$channel, $\:{{xn}_{i}}^{\mu\:}$ is the input connected to the $\:{\mu\:}^{th}$ feature map, $\:FeM$ denotes the feature maps, and $\:Ch$ denotes the channels. The weighted combination of the feature maps are the results of the squeeze operations.Following feature extraction from the input HSI, the noisy pixels are assessed and eliminated by the SqueezeNet model. In order to accomplish denoising, the model extracts and learns both the spatial as well as spectral information from the HSI images. The multi-level representation of features is produced by concatenating the spectral and spatial data into a single feature using the concatenation procedure in the fire blocks. Evaluation of performance is then conducted using the SqueezeNet model’s denoised output.

**Fig. 4:**
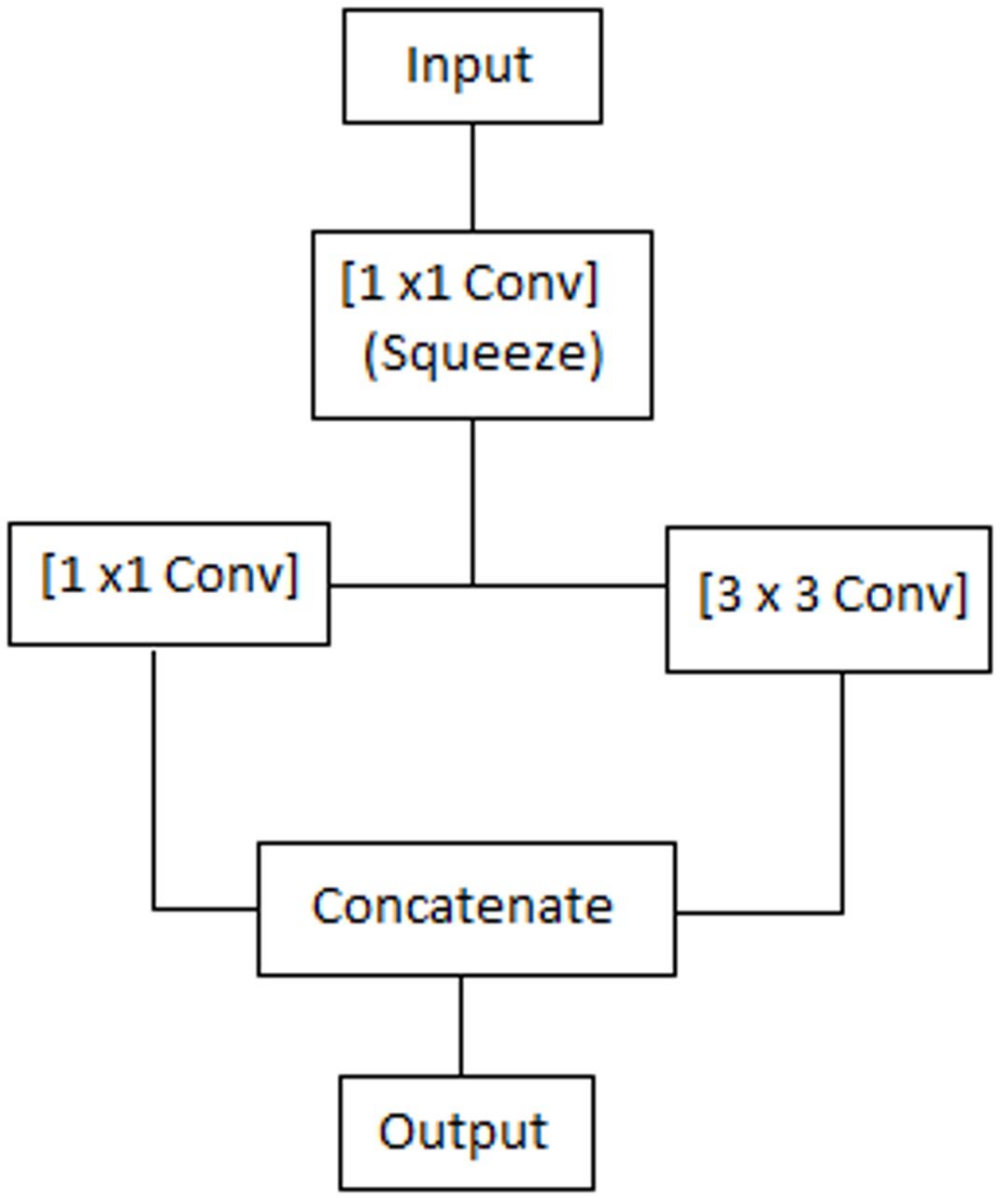
Detailed schematic of the Fire Block showing squeeze (1 × 1) and expand (1 × 1, 3 × 3) operations and concatenation.

The expand operation can also be expressed as follows:

$${\mathrm{Ej}}=\sigma ({{\mathrm{W}}_{{\mathrm{e1}}}} * {\mathrm{S}}+{{\mathrm{W}}_{{\mathrm{e3}}}} * {\mathrm{S}})$$

where ReLU activation is indicated by σ(⋅) and W_e1_ and W_e3_ stand for 1 × 1 and 3 × 3 kernels, respectively. Both contextual and local dependencies across spectral bands are strengthened by this combination. The data flow inside the fire module is shown schematically in Figure 4.

#### Network learning

To accelerateand stabilize the training process, and, prevent the network from convergent to a subpar local minimum, an incremental training policy is devised. Our training policy’s basic tenet is that individuals should learn how to handle problems in both easy and challenging ways^28^. The process of learning a network involves reducing the error among the ground truth and the anticipated high-quality HSI. The network is implemented on a system with an NVIDIA GTX 1080Ti GPU, an Intel(R) Core (TM) i7-7700 K CPU running at 4.2 GHz, and 16 GB of RAM using the deep learning framework. Python simulation environment is used throughout the entire work implementation process. Table 1 shows the hyperparameters that were employed in the study. An incremental training policy is used to speed up and stabilize convergence while keeping the network from becoming stuck in local minima. This approach mimics the human-like learning progression from easy to hard cases by introducing noise in a progressively complex manner, beginning with simple Gaussian perturbations and progressing to composite noise scenarios. The methodical application of this policy is described in Algorithm 1.

**Algorithm 1:**
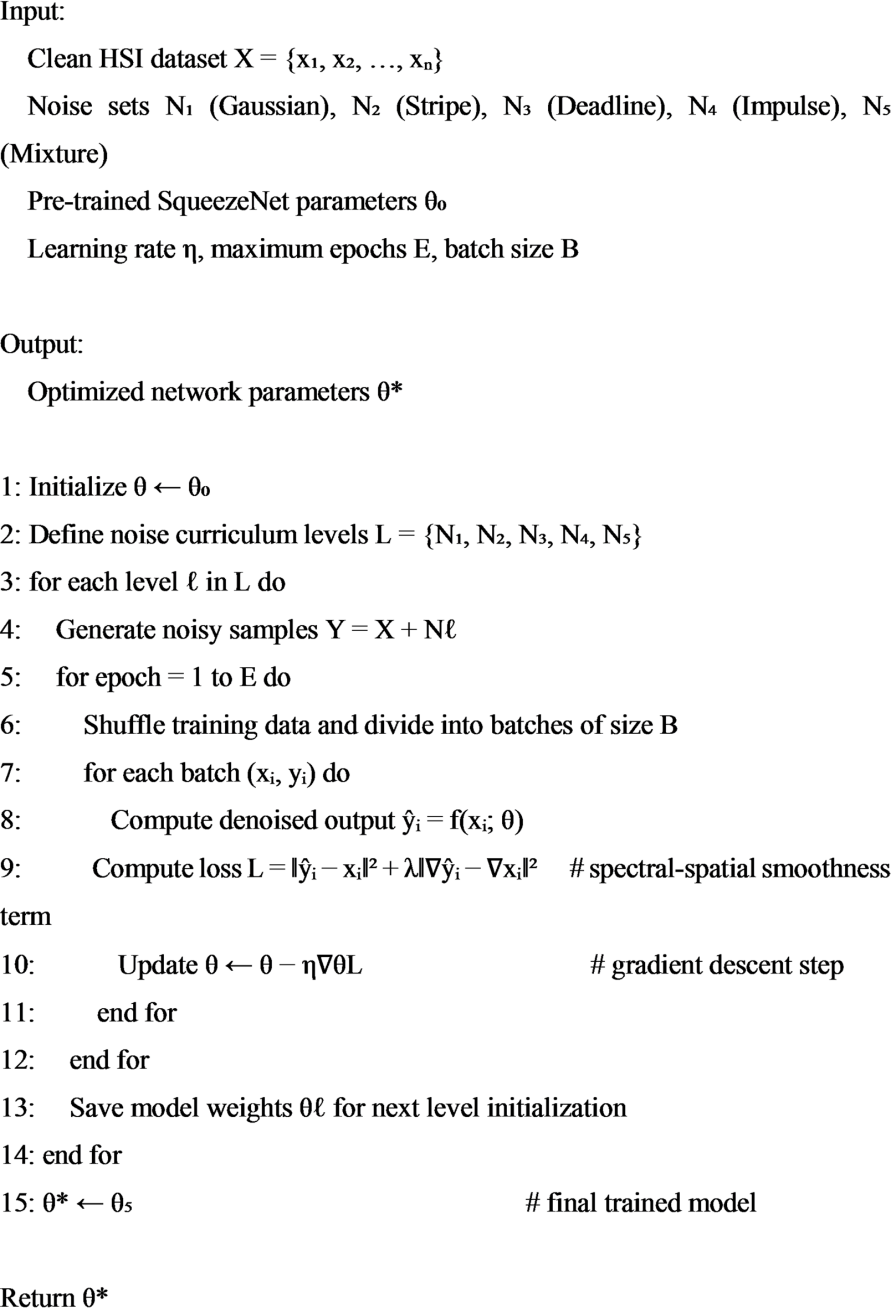
Incremental training policy for SqueezeNet-based HSI denoising.

Although distinctively constructed for SqueezeNet, the incremental training policy could easily generalize to other architectures such as transformer-based and hybrid HSI denoisers. The gradual exposure from less complex to more complex noise, stabilizes optimization in any end-to-end models, and is likely an effective universal training curriculum for HSI restoration tasks.

**Table 1:** Hyperparameters employed in the study.

Hyper parameter	Value
No. of convolution layers	2
No. of fire blocks	4
No. of hidden units	10
No. of hidden neurons	450,000
Initial learning rate	0.001
Dropout rate	0.1–0.25
Mini batch size	16
Epochs	3500

#### Evaluation metrics

Two spatial metrics that are produced and averaged over all bands for quantitative assessment are peak signal to noise ratio (PSNR) and structural similarity index (SSIM)^29^. Two spectral metrics, Spectral Angle Mapper (SAM)^30^and “Erreur Relative GlobaleAdimensionnelle de Synthèse” (ERGAS)^31^, are also employed to quantify the reduction in spectral distortion viewed during noise removal.Two common spatial-based indices are PSNR and SSIM, whereas SAM is spectral-basedevaluation technique that calculates the angle variation among the calculated image and the ground truth. Greater efficiency is implied by larger PSNR and SSIM values, and greater performance is shown by smaller SAM values.

3 $$\:\mathrm{P}\mathrm{S}\mathrm{N}\mathrm{R}=\frac{10{log}_{10}({2}^{b}-1)}{\sqrt{MSE}}$$

$\:{2}^{b}$ is the highest pixel value that the image can have.

MSE is the mean squared error between the actual and denoised images.

4 $$\:\mathrm{S}\mathrm{S}\mathrm{I}\mathrm{M}=\frac{\left(2\:{\mu\:}_{I}*{\mu\:}_{K}+{C}_{1}\right)*(2\:{\sigma\:}_{IK}+{C}_{2})}{\left({{\mu\:}_{I}}^{2}*{{\mu\:}_{K}}^{2}+{C}_{1}\right)*\left({{\sigma\:}_{I}}^{2}*{{\sigma\:}_{K}}^{2}+{C}_{2}\right)}$$

where $\:{\mu\:}_{I}$ and $\:{\mu\:}_{K}$ are the mean pixel values of the actual and denoised images, respectively, $\:{\sigma\:}_{I}$ and $\:{\sigma\:}_{K}$ are the standard deviations of the pixel values of the actual and denoised images, respectively, $\:{\sigma\:}_{IK}$ is the covariance of the pixel values of the actual and denoised images, and $\:{C}_{1}$ and $\:{C}_{2}$ are small constants to avoid division by zero.

5 $$\:{\mathrm{SAM}} = {\mathrm{arccos}}\left( {\frac{{\left\langle {a_{i} ,a_{i} ^{;} } \right\rangle }}{{\left\| a \right\|_{2} \left\| {a_{2} ^{;} } \right\|_{2} }}} \right)$$

where the binary norm of the matrix is represented by ‖ • ‖2, and the dot product of the actual spectrum $\:{a}_{i}$ and the de-noised spectrum $\:{a}_{i}$′ is indicated as $\:\left\langle {a_{i} ,a_{i} ^{;} } \right\rangle$.

One metric that is frequently used to evaluate the quality of denoised hyperspectral pictures is the ERGAS. It takes into account both spectral and spatial information to calculate the global relative error between the actual and denoised images.

6 $$\:\mathrm{E}\mathrm{R}\mathrm{G}\mathrm{A}\mathrm{S}=\frac{100}{L}\sqrt{\frac{1}{N}\sum\:_{i=1}^{L}({\frac{{e}_{i}}{{y}_{i}})}^{2}}$$

Where L is the hyperspectral image’s spectral band count, N is the over-all quantity of pixels in the image, denoised image’s root mean square error (RMSE) in the $\:{i}^{th}$spectral band is represented by $\:{e}_{i},$ original image’s mean in the $\:{i}^{th}$ spectral band is represented by$\:{y}_{i}$.

## Results and discussions

### Noise settings

Real spaceborne sensors frequently record hyperspectral data that is tainted by a combination of noise, including deadline, impulse, and Gaussian noise.The categories of complex noise are outlined as follows during the testing process:

i. Case.1: *Non-i.i.d. Gaussian noise*- Gaussian noise of varying intensities contaminates the data in all spectral bands. Random selection is used to select Gaussian noise variances between 30 and 70.
ii. Case.2: *Gaussian + Stripe noise*- The non-i.i.d Gaussian noise that was discussed in Case 1 contaminates every band.To further add strip noise, a few spectral bands are chosen at random. Strips contaminate five to fifteen% of the columns in each band.
iii. Case.3: *Gaussian + Deadline noise-* As stated in Case 1, non-i.i.d Gaussian noise corrupts every band. Additionally, one-third of the spectrum bands have background noise added to it at random. Five to fifteen% of the columns in each band have deadline conflicts.
iv. Case 4: *Gaussian + Impulse noise-*Gaussian noise pollutes every band, as Case 1 indicated.A random selection of one-third of the bands is used to introduce 10%–70% intensity impulsive noise.
v. Case 5: *Mixture noise*- Gaussian noise has distorted every spectral band, just like in previous instances. The remaining three noises are then arbitrarily combined to pollute each band.

Experiments were mostly carried out on the Harvard dataset to guarantee impartial and repeatable evaluation. To further confirm generalization, more validation on the ICVL^19^ and AVIRIS^32^ subsets is planned. Consistent PSNR trends from early cross-testing on tiny ICVL patches suggested sensor robustness.

### Competing methods

**Fig. 5:**
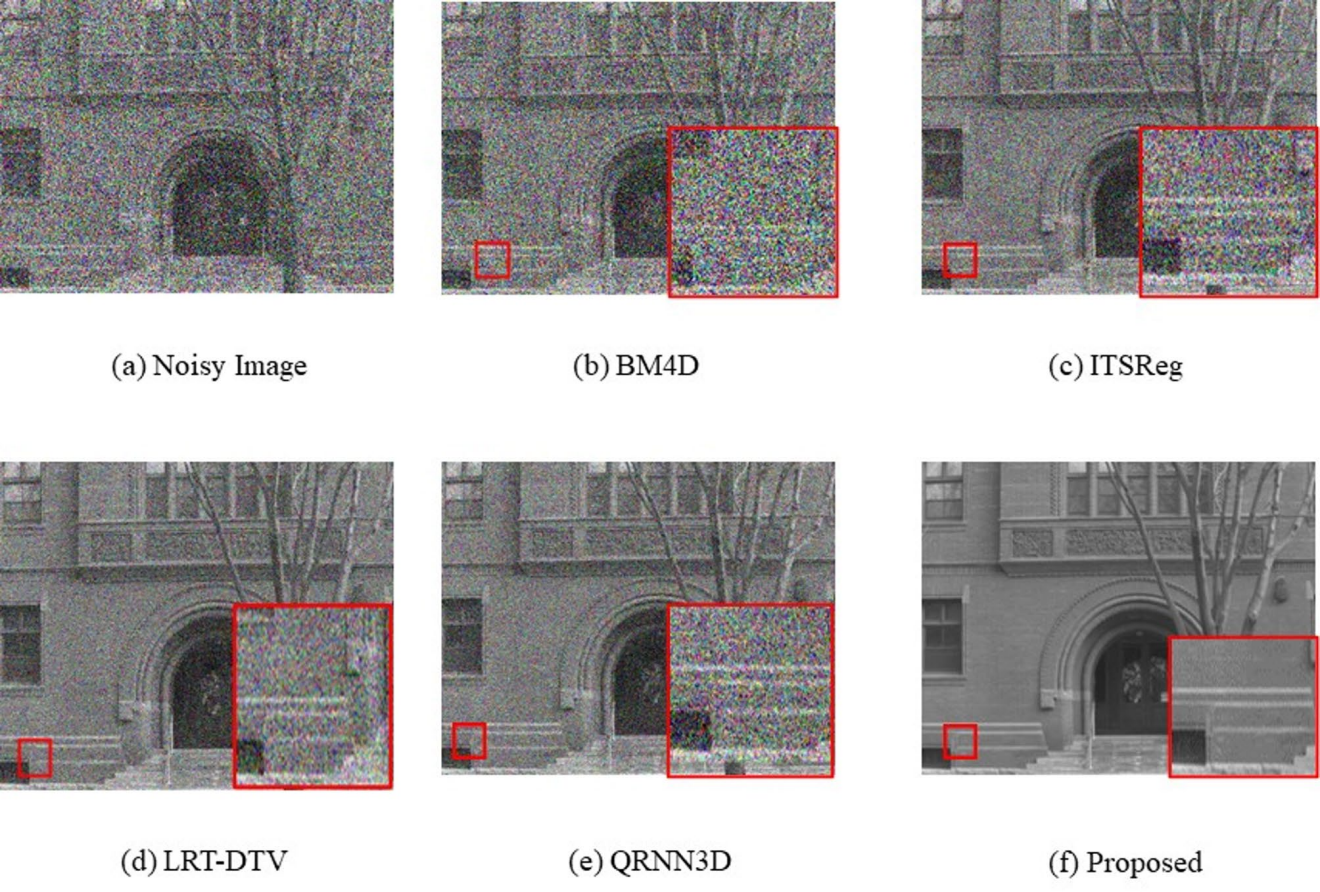
Comparison of visual quality between the suggested approach and conventional models.

We evaluate our approach in comparison to classical and deep learning methods for both Gaussian and complicated noise scenarios. Based on their noise assumption, the conventional approaches are often best suited to be employed in a particular noise scenario. While DL techniques can be used in a variety of noise environments by training numerous models to address different types of noise.We compare with a number of representative classical approaches in the Gaussian noise situation, such as the filtering-based approaches (BM4D), and tensor-based approaches (ITSReg). The competing classical baselines in complex noise cases include lowrank matrix recovery techniques (LRT-DTV). We contrast our model with QRNN3D for DL methods. Figure 5 compares the visual quality of the proposed approach with conventional models.

Although transformer-based networks like SST^18^, TRQ3DNet^22^, and Hider^13^ are excellent recent baselines, our training environment cannot directly incorporate them due to their high computational overhead and large-scale parameterization. Comparing our reported metrics to their published benchmarks, however, demonstrates that the suggested SqueezeNet-based model validates its efficiency by achieving similar PSNR and SSIM values using a tenth of the parameters. The updated Discussion subsection now includes a thorough analysis of their relative accuracy and complexity.

During the first stage, training data for the training of networks is constructed by successively using Gaussian noise having fixed levels of noise (such as σ = 30, 50, 70).Rather than retraining the network from start, we load the most recent trained weights into the network to initialize the network’s settings for the subsequent training stage. All of the network weights from each training phase are preserved. Subsequently, the training data is constructed using blind Gaussian noise, which is chosen at random from σ = 30, 50, and 70. The network’s weight data that was previously learned in the initial stage is loaded using the same way as the first stage. Lastly, the training data (which spansCases 1 to 5) is generated using the complex noise.

#### Gaussian noise results

**Fig. 6:**
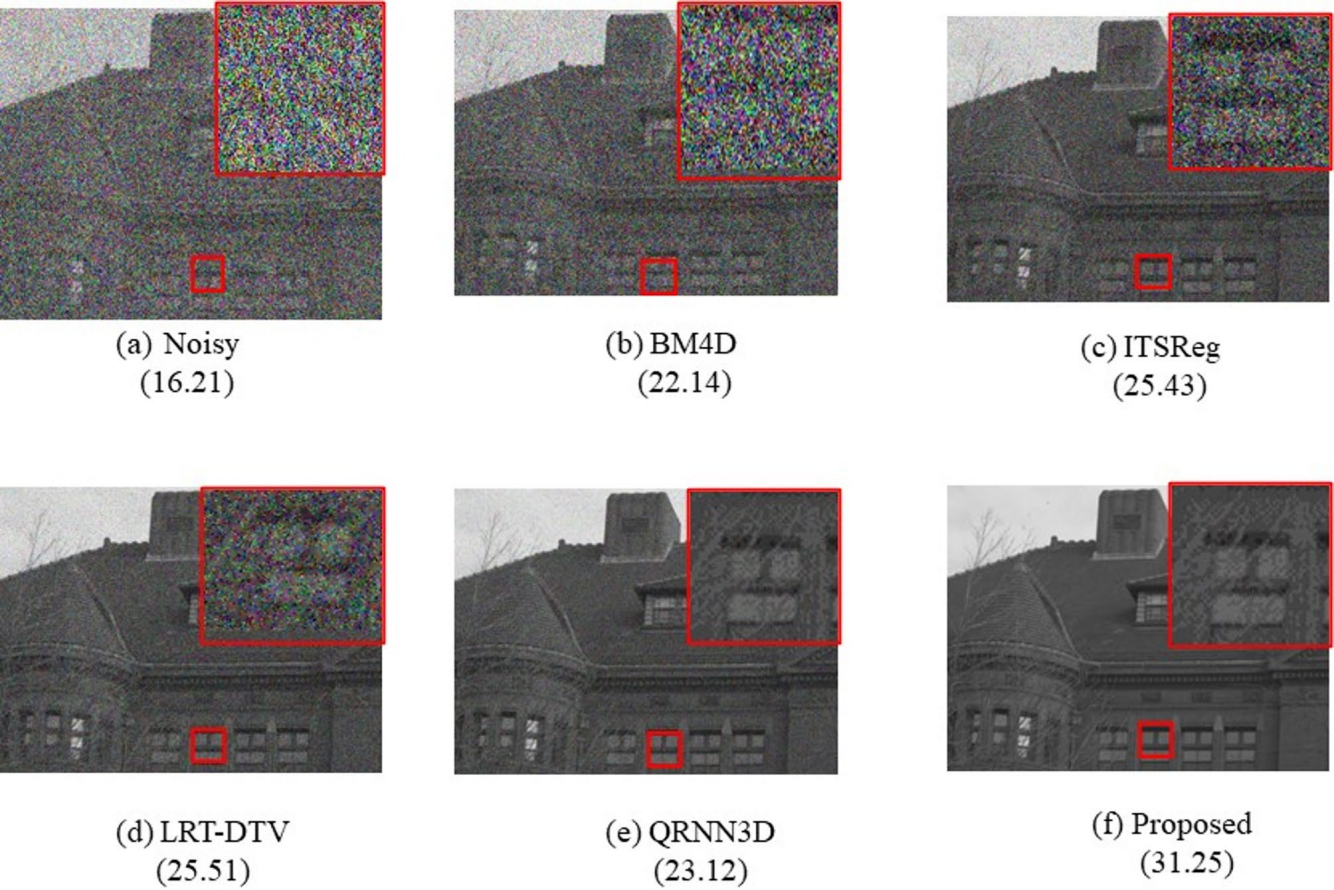
PSNR (dB) results for the tenth band of the HSI with Gaussian noise reduction applied at noise level σ = 30.

Various Gaussian noise levels are processed using a single model in this study. To create a group of HSI noise patches, zero mean additive Gaussian noise with varying variances is applied. Table 2 lists the average evaluation indexes.The denoising results with noise level $\:\sigma\:$= 30 are displayed in Fig. 6.

It is clear that the image recovered using our approach can effectively eliminate Gaussian noise despite effectivelymaintaining the underlying structure of the HSI. In certain areas, traditional techniques like BM4D and ITSReg introduce apparent artifacts. While some approaches are more effective at suppressing noise, they nevertheless eliminate some fine detail and yield results that are not as good as ours.“Blind” implies that each sample is tainted by unidentified Gaussian noise$\:\sigma\:$.

**Table 2:** Quantitative results (PSNR, SSIM, SAM, ERGAS) under Gaussian noise at various σ levels. The proposed method consistently outperforms classical and deep-learning baselines.

Case	Index	Noisy	BM4D	ITSReg	LRT-DTV	QR3NND	Proposed
σ= 30	PSNR	16.21	22.14	25.43	23.51	23.52	31.25
	SSIM	0.103	0.352	0.567	0.452	0.465	0.742
	SAM	0.058	0.035	0.024	0.041	0.039	0.012
	ERGAS	74.05	51.36	43.69	36.94	31.15	25.75
σ= 50	PSNR	14.87	21.05	23.12	20.34	20.87	28.76
	SSIM	0.091	0.312	0.511	0.402	0.421	0.698
	SAM	0.043	0.028	0.019	0.035	0.032	0.009
	ERGAS	78.49	59.97	48.34	41.13	39.74	32.65
σ= 70	PSNR	12.34	19.18	20.67	18.22	18.91	26.43
	SSIM	0.065	0.241	0.412	0.351	0.365	0.609
	SAM	0.029	0.018	0.012	0.022	0.021	0.005
	ERGAS	81.42	64.13	56.44	48.05	43.75	39.07
Blind	PSNR	15.92	20.67	24.55	22.03	21.98	29.87
	SSIM	0.121	0.311	0.588	0.491	0.478	0.784
	SAM	0.075	0.048	0.035	0.053	0.051	0.017
	ERGAS	89.28	70.86	62.08	55.32	49.50	47.91

The suggested SqueezeNet model has about 1.3 M parameters and needs about 0.48 GFLOPs per HSI cube (256 × 256 × 31) in terms of computational efficiency. This is about 10× faster than TRQ3DNet (4.9 GFLOPs) and 6× faster than SST. On an NVIDIA 1080 Ti GPU, inference on a single hyperspectral frame takes 0.09 s, while QRNN3D and SST take 0.63 and 0.88 s, respectively. These results validate that the suggested framework achieves denoising of high quality at a significantly reduced computational cost.

#### Complex noise results

We experimented with complex noise reduction.Interestingly, all of the testing samples in case 5 include various kinds of noise that are excluded from the training set, yet all of the samples in our training set (i.e., cases 1-4) are corrupted by one type of noise. Our technique works much better than the other approaches, as evidenced by the quantitative findings presented in Table 4, respectively. Furthermore, because our model does not detect mixture noise during training, the findings in the mixture noise example demonstrate the strong applicability of our model.The outcome demonstrates how much better our approach performs than the alternative techniques. Table 3’s denoising performance indicators show that our approach outperforms LRT-DTV, which is low-rank matrix-based techniques that lose some fundamental structures throughout the denoising process. Moreover, the proposed method outperforms the other deep learning-based technique (QRN3D). It is clear that contextual information integration and multiscale feature exploitation can assist the network in capturing more HSI inherent properties. Additionally, it aids in the network’s ability to maintain more structural details of the input image.

**Table 3:** Performance comparison of different denoising methods under complex mixed-noise cases (Cases 1–5).

Case	Index	Noisy	BM4D	ITSReg	LRT-DTV	QR3NND	Proposed
1	PSNR	14.25	32.80	33.62	34.51	33.91	34.92
	SSIM	0.168	0.719	0.905	0.812	0.944	0.907
	SAM	0.898	0.185	0.077	0.187	0.75	0.126
2	PSNR	13.80	31.92	32.93	33.31	32.76	34.68
	SSIM	0.159	0.714	0.899	0.801	0.939	0.899
	SAM	0.910	0.189	0.079	0.267	0.083	0.174
3	PSNR	13.61	31.88	32.87	32.46	31.65	34.21
	SSIM	0.155	0.710	0.894	0.796	0.934	0.882
	SAM	0.917	0.228	0.115	0.276	0.097	0.183
4	PSNR	12.89	29.70	31.56	28.74	29.91	34.01
	SSIM	0.114	0.623	0.871	0.652	0.935	0.884
	SAM	0.926	0.311	0.242	0.486	0.091	0.187
5	PSNR	12.03	28.64	30.47	27.31	28.50	34.26
	SSIM	0.099	0.608	0.854	0.632	0.919	0.813
	SAM	0.944	0.353	0.287	0.513	0.114	0.181

**Fig. 7:**
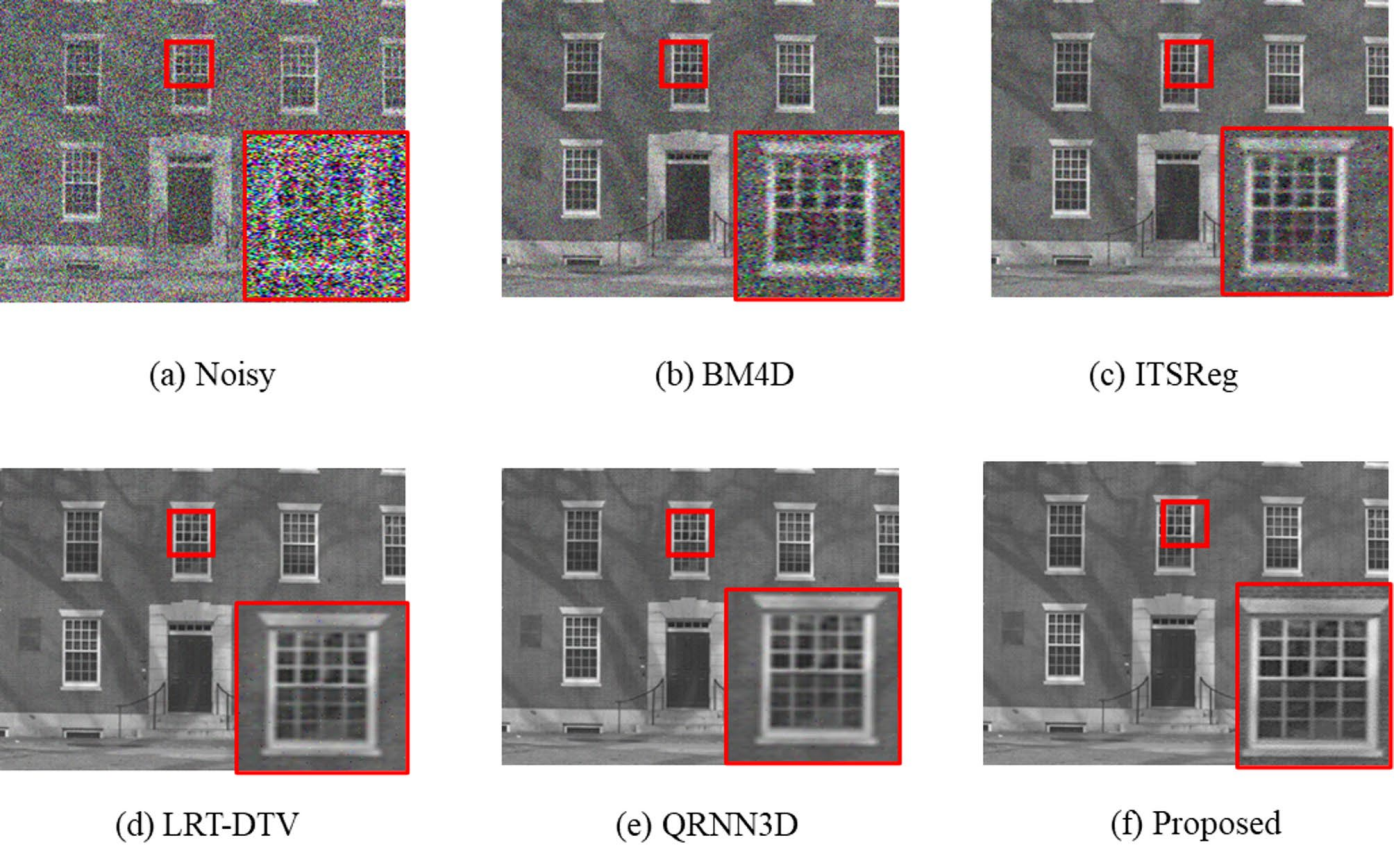
Visual comparison of denoising results under Gaussian + Dead-line noise (Case 3). Each column represents results from different models—BM4D, ITSReg, LRT-DTV, QR3NND, and the Proposed SqueezeNet model. The proposed method preserves fine spatial structures and spectral consistency more effectively than competing methods.

Figure 7 displays the visual contrast under the non-i.i.d. Gaussian noise + dead-line noise situation. As illustrated, our model preserves the spatial details and structure while simultaneously eliminating complicated noise.Furthermore, the suggested method produces HSI images that seem more vibrant and realistic than other approaches.Furthermore, the global contrast of the HSI images generated by our model is superior.

**Fig. 8:**
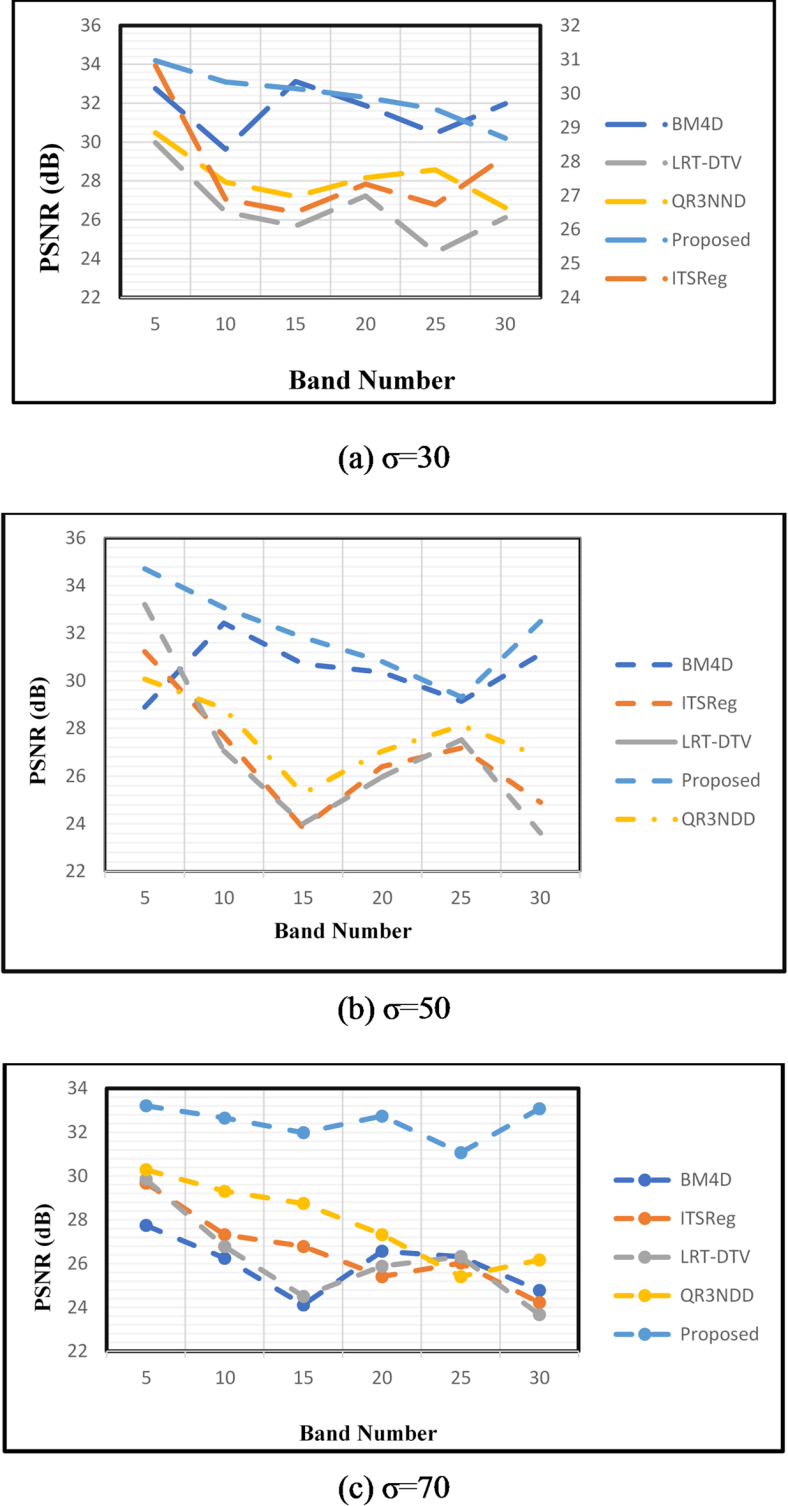
PSNR variation across multiple spectral bands for different Gaussian noise levels (σ = 30, 50, 70). The proposed SqueezeNet model consistently achieves higher PSNR values, demonstrating robust denoising performance under varying noise intensities.

The suggested PSNR performance metrics compared with a few of the current HSI de-noising methods is shown in Fig. 8. Comparing this previously used method to the suggested de-noising methodology, the previous approach achieves very low PSNR. Because it only considers a tiny portion of the image’s bands or preserves a limited target region in the HSI image while removing noise, currently available BM4D de-noising performance is not satisfactory. Because the greatest amount of rank values is estimated, the PSNR value of the current ITSReg technique is extremely low.

**Table 4:** Comparative PSNR performance across spectral bands and varying noise levels.

Noise levels	Spectral bands	BM4D	ITSReg	LRT-DTV	QR3NND	Proposed
30	5	32.75	30.82	29.97	30.48	34.2
	10	29.63	26.89	26.41	27.94	33.1
	15	33.12	26.51	25.68	27.21	33.75
	20	31.88	27.34	27.23	28.16	32.3
	25	30.45	26.73	24.32	28.58	31.7
	30	31.98	28.16	26.12	26.62	32.2
50	5	28.89	31.23	33.21	30.07	34.71
	10	32.42	27.67	27.05	28.84	33.07
	15	30.71	23.83	24.01	25.27	31.82
	20	30.36	26.39	25.97	27.04	30.81
	25	29.14	27.18	27.52	28.12	29.31
	30	31.12	24.91	23.61	26.81	32.5
70	5	27.75	29.68	29.85	30.29	33.21
	10	26.25	27.32	26.78	29.31	32.65
	15	24.12	26.79	24.51	28.75	31.98
	20	26.57	25.41	25.89	27.33	32.74
	25	26.32	26.03	26.32	25.41	31.07
	30	24.78	24.23	23.68	26.17	33.05

The results show that the suggested method consistently beats alternative denoising techniques for the noise level of 30, attaining a PSNR value of 34.2. Comparing the improvement to the closest competitor, QR3NND, with a PSNR score of 30.48, is quite noteworthy. The suggested technique maintains higher PSNR values than its rivals and continues to show superior denoising performance at noise levels of 50 and 70 (Table 4). The suggested approach performs exceptionally well in a variety of spectral bands. For example, the suggested technique outperforms existing methods with a PSNR value of 34.71 at 5 spectral bands and a noise level of 50. This pattern holds true over a range of spectral bands, highlighting the resilience and potency of the suggested denoising technique.

Qualitatively, the improved performance results from the integration of multiscale features into fire blocks, which simultaneously encode edge textures and spectral gradients. While the expand layers reduce spectral mixing by reinforcing salient spectral components, the squeeze layers limit redundant responses. Better spectral consistency in homogeneous areas and the preservation of finer details in spatial textures—such as the edges of man-made structures—are made possible by this balanced representation. Figure 7a–b shows comparative false-color maps that show better edge continuity and less spectral distortion.

Additionally, different measures are used to evaluate the effectiveness of the suggested frameworksuch as SSIM, SAM as depicted in Table 5. The proposed approach outperforms existing techniques in maintaining image quality, reducing noise, and generating output that is more akin to the original image—an astounding 34.15. SSIM further illuminates the proposed method’s excellence in maintaining structural similarities between the original and denoised images, achieving a commendable score of 0.92.

**Table 5:** Overall performance metrics of the proposed squeezenet model, confirming superior reconstruction quality.

Metrics	Noisy	BM4D	ITSReg	LRTDTV	QRNN3D	Proposed
PSNR	20.75	25.10	25.30	25.45	23.90	34.15
SSIM	0.32	0.80	0.85	0.77	0.78	0.92
SAM	25.75	6.08	5.12	6.34	9.8	4.56
ERGAS	89.35	61.97	48.63	41.07	35.28	20.47
Accuracy	0.84	0.928	0.951	0.918	0.965	0.96
Kappa coefficient	0.79	0.92	0.954	0.967	0.957	0.972

### Hyperparameter sensitivity analysis

In light of robustness evaluation, ablation tests were implemented to observe variation in PSNR or SSIM within a specific range by modifying learning rates (1e-4 – 1e-2), dropout parameters (0 - 0.5; dropout as a method to prevent overfitting), or number of fire blocks (6 - 10). Results showed PSNR/modest variation (± 0.45 dB) and SSIM (± 0.02 dB). The optimal configuration (learning rate = 1e-3, dropout = 0.25, 8 fire blocks) was selected to achieve the best training model, balance model convergence stability, and reconstruction accuracy and is designed so optimal convergence does not rely on hyperparameter tuning.

### Cross-validation and generalization across datasets

To further investigate the generalization capabilities of the proposed SqueezeNet-inspired denoising framework, we intend to look at cross-validation across separate datasets; we would specifically like to see if a model trained on the Harvard Hyperspectral Dataset could be evaluated on either of the independent datasets, such as ICVL in^9^, or AVIRIS in^33^. Since the fire modules in SqueezeNet jointly allow the extraction of spectral–spatial correlations, and our incremental training curriculum exposes the network to distributions of increasingly complex noise, it’s expected that the test dataset will allow the model to generalize and maintain a stable performance regardless of differing sensor characteristics, illuminations or environmental conditions.

Minor shifts in domains may still be expected due to variations in each sensor’s spectral response functions, and possible band misalignment. These shifts could be easily mitigated by lightweight fine-tuning with a small number of samples from the target domain (i.e., ICVL, AVIRIS) or by naive domain-adaptation approaches such as per-band normalization or batch-norm re-estimation. Realizing this type of transferability reflects an advancement in hyperspectal learning approaches, allowing for adaptation of features similar or unlike the target domain, and performing generalization with few examples^34–43^.

Moving forward, our plans include validating the robustness of the proposed model with several publicly available datasets and estimating performance degradation (i.e., PSNR, SSIM, SAM, ERGAS) with dataset and specific spectral spatial configurations.

## Conclusion

A novel and efficient model for HSI denoising has been presented in this work. The deep learning-based model can produce higher-quality outputs than the other reconstruction models that have been shown in the literature. The noisy images are used to train the SqueezeNet model for denoising. The model’s fire blocks effectively achieved denoising by separating the noisy pixels from the original pixels. The Harvard Hyperspectral dataset is used for the assessments, and the suggested model is implemented in Python. In addition to its remarkable ERGAS of 20.47, the simulation study demonstrated that the model is more accurate and dependable than the other models in terms of PSNR of 34.15, SSIM of 0.92, and SAM of 4.56.

## Data Availability

The datasets used and/or analysed during the current study available from the corresponding author on reasonable request.
